# Development and Evaluation of a Novel Taqman Real-Time PCR Assay for Rapid Detection of *Mycoplasma bovis*: Comparison of Assay Performance with a Conventional PCR Assay and Another Taqman Real-Time PCR Assay ‡

**DOI:** 10.3390/vetsci2010032

**Published:** 2015-03-16

**Authors:** Hemant Naikare, Daniela Bruno, Debabrata Mahapatra, Alesia Reinisch, Russell Raleigh, Robert Sprowls

**Affiliations:** Texas A&M Veterinary Medical Diagnostic Laboratory, 6610 Amarillo Blvd West, Amarillo, TX 79106, USA; E-Mails: danirbruno@gmail.com (D.B.); debabrata77@gmail.com (D.M.); areinisch@tvmdl.tamu.edu (A.R.); r-raleigh@tvmdl.tamu.edu (R.R); r-sprowls@tvmdl.tamu.edu (R.S.)

**Keywords:** *Mycoplasma bovis*, cattle, *Taqman* real-time PCR, *uvrC* gene

## Abstract

The objective of this study was to develop and validate a Taqman real-time PCR assay for the detection of *Mycoplasma bovis* (*M. bovis*). Unique primers targeting the highly conserved house-keeping gene (*uvrC*) were designed and the probe sequence was derived from a previously published microarray study. There was 100% agreement in the outcome between our assay and the other two published assays for *M. bovis* detection. The analytical limit of detection of our assay is 83 copies of the *uvrC* gene. This assay was validated on a total of 214 bovine clinical specimens that were submitted to the Texas A&M Veterinary Medical Diagnostic Laboratory (TVMDL), Texas, USA. The specificity of the assay was assessed to be 100% since no cross-reactivity occurred with 22 other bacterial and other *Mycoplasma* species. We conclude that the *uvrC* gene serves as a good and reliable diagnostic marker for the accurate and rapid detection of *M. bovis* from a wider variety of specimen matrices.

## 1. Introduction

*Mycoplasma bovis* (*M. bovis*) continues to be a significant economic burden to the beef and dairy cattle industries. It is a major bovine mycoplasma pathogen associated with pneumonia, mastitis, arthritis, and abortion. It has been reported that *M. bovis* accounts for monetary losses of about 150 million euros across Europe and more than $100 million/year in the US due to mastitis and respiratory disease [1].

*M. bovis*, which belongs to the family *Mycoplasmataceae* in the class Mollicutes, is characterized by its small genome size (580–1380 kb), devoid of cell wall, and require complex growth media [2]. *M. bovis* is widespread within the bovine population in enzootically infected areas. Infection is usually introduced into *M. bovis*-free herds by asymptomatic calves or adult carrier cattle that are actively shedding the organism. Organisms can be shed via the respiratory tract for many months or even years, and such animals act as a reservoir of infection. Contact animals become infected via the respiratory tract, the teat canal, genital tract or artificial insemination with infected semen [1].

Infections caused by *M. bovis* are difficult to treat by antibiotic therapy. Their lack of cell wall makes them resistant to antimicrobials that target the cell wall such as beta-lactams [1,3]. Control methods rely mainly on culling infected animals and testing newly purchased animals before addition to the herd and maintaining extensive hygiene within the premises [4]. Hence, rapid and reliable diagnostic tools are in constant demand to detect infected animals and control the disease caused by *M. bovis*.

In comparison to the other bacterial pathogens that affect cattle, *M. bovis* are slow growers, and their fastidious growth requires the addition of sterols. It may take 3 to 10 days to grow at 37 °C in presence of 5%–10% CO_2._ They produce “centered” colonies that give a “fried-egg” like appearance [5]. Although scientific literature cites culture as the gold standard method for detection of *M. bovis*, culture offers limited sensitivity and specificity due to multiple factors [1,6,7]. Serological tests determine previous exposure to *M. bovis* and are considered to be more sensitive than culture in diagnosing chronic infections. Different types of ELISA tests with whole cell or treated antigen have been described to detect antibodies in milk and serum and are also commercially available. Antibodies to *M. bovis* can be detected with various immunological methods including counter current electrophoresis, ELISA, immunoblotting, immunobinding and immunohistochemistry methods [8]. Although serology can help in identifying mycoplasma negative herds, the presence of high sero-prevalance in many populations limits the utility of serological tests [7].

The complete genome sequence of the international reference strain of *M. bovis* PG45 (ATCC 25523) has been reported [9]. Several PCR based molecular tests have been published which allow *M. bovis* identification using conventional, SYBR, Taqman real-time PCR or loop-mediated isothermal amplification (LAMP) platforms with varying sensitivity and specificity indices [10,11,12,13,14,15]. The known diagnostic targets for *M. bovis* detection primarily include 16S *rRNA* and *uvrC* genes. The *uvrC* gene is a highly conserved housekeeping gene that encodes for a DNA repair enzyme “deoxyribodipyrimidine photolyase” [10]. Subramaniam and colleagues have demonstrated that although the *uvrC* gene is well conserved in *M. bovis* and in another small ruminant pathogen *Mycoplasma agalactiae*, this gene differs significantly between the two phylogenetically closely related *Mycoplasma* species [10]. Thomas and colleagues [16] have demonstrated the *uvrC* gene to be a conserved target suitable for PCR based diagnostics by PCR amplification and sequencing from ninety-two and twenty *M. bovis* strains respectively. Interestingly, Marenda and colleagues [17] have demonstrated the limitation of *uvrC* target’s universality for *M. bovis* detection since the *uvrC* gene sequences are only slightly divergent making it difficult to differentiate *M. bovis* and *M. agalactiae*. Based on their suppression subtractive hybridization study, Marenda and colleagues have further proposed that the *polC* target based PCR should be included as a complement to the *uvrC* target based detection of *M. bovis* [17]. Other diagnostic markers that have been reported for PCR based identification of *M. bovis* include an ATP binding protein *oppD*/*F* and membrane protein 81 [7].

Our objective was to develop and evaluate a rapid and accurate *uvrC* gene-based Taqman real-time PCR assay for the detection of *M. bovis* in a wide variety of clinical specimen matrices that can be utilized for routine diagnostic testing, molecular surveillance, and export testing.

## 2. Experimental Section

### 2.1. Clinical Specimens

A total of 214 specimens comprising milk samples: individual animals and bulk tanks (78); fresh lungs (45); joint fluids (5); semen (8); and nasal swabs (78) submitted to the TVMDL laboratory for culture and/or *M. bovis* PCR were included in this study. These specimens originated from dairy farms, beef feed lots, and from bison farms across Texas and other states in the US.

### 2.2. DNA Extraction

The clinical specimens were prepared for nucleic acid purification as follows: (i) milk: ~25 to 50 mL of milk sample was pelleted by centrifuging at 3000–4000 g for 15–20 min. The supernatant fat and excess liquid were removed, and the pellet was washed twice with sterile phosphate buffered saline (PBS). The washed pellet was re-suspended in 200 µL of sterile PBS and then used for nucleic acid purification; (ii) Lung tissues: ~1–2 grams of tissue was aliquoted and mixed with 10–20 mL of sterile PBS and homogenized using a stomacher (10% w/v) (Stomacher^®^ Lab Blender Model 8.0 Seward Medical Limited, London-UK 10/94). Following disruption, 200µL of the tissue supernatant was used for nucleic acid purification; (iii) Nasal swab samples were directly re-suspended in ~ 400 to 500 µL of sterile PBS and 200 µL of it was used for nucleic acid extraction; (iv) semen and joint fluid specimens: 200 µL of specimen was directly used for nucleic acid extraction; (v) *Mycoplasma* sp. isolates/colonies: ~1–4 colonies were transferred into 200 µL of sterile PBS using sterile disposable loop, and subjected to nucleic acid purification.

The nucleic acid purification from 200 µL of processed clinical specimens was performed using QIAamp DNA mini kit (Qiagen #51306) as per the manufacturer’s instructions. DNA was eluted in 100 µL of sterile water and stored at −20 °C until the PCR amplification could be performed.

### 2.3. TaqMan Real-Time PCR Assay

The *M. bovis uvrC* gene sequence was retrieved from the Genbank accession # AF003959.1. *M. bovis* species specific primers were designed using Primer3 software (http://frodo.wi.mit.edu/). The probe sequence was derived from a previously published microarray study [18]. The primers and probe were synthesized from IDT-DNA and Life Technologies respectively, and the sequence information is listed in Table 1.

**Table 1:** Sequences of primers and probes used in this study.

Gene Target	Oligo	Sequence (5'→3')	Reference	Product Size
*uvrC*	Forward Primer	GAG AAT GCT TCA GTA TTT TGA CGG	This study	170 bp
	Reverse Primer	CAA AAG CAA AAT GTT AAA TTC AGG	This study	
	Probe	(6 FAM) CAT ATA TAA GTG AGA CTA ACT TAT T(MGB)	[18]	
*uvrC*	F2024	TCT AAT TTT TTC ATC ATC GCT AAT GC	[12]	112 bp
	R2135	TCA GGC CTT TGC TAC AAT GAA C		
	M.bov	(FAM) AAC TGC ATC ATA TCA CAT ACT (MGB)		
16S rRNA	Forward Primer	CCT TTT AGA TTG GGA TAG CGG ATG	[19]	360 bp
	Reverse Primer	CCG TCA AGG TAG CAT CAT TTC CTA T		

Taqman real-time PCR was carried out in Applied Biosystems 7300 or 7500 Fast thermal cycler instruments (Applied Biosystems, Foster City, CA, USA) in a standard mode. The PCR was performed in a 20 µL final reaction volume. The 3 µL of template DNA was added to 17 µL master mix prepared with TaqPCR core kit (Qiagen #201225) containing 2.0 µL 10X PCR buffer, 0.4 µL dNTP (10 mM each), 2.4 µL·MgCl_2_ (25 mM), 0.8 µL each of forward and reverse primers (25 µM), 0.8 µL of probe (7.5 µM), 0.25 µL Taq DNA Polymerase (5 units/µL) and 9.55 µL of water. The PCR assay was performed using 96-well plates that were sealed with optical sealing tape (Applied Biosystems, Foster City, CA, USA). Reaction mixtures containing water substituted for DNA templates were used as negative controls. The thermocycling conditions were set as per the following parameters: 95 °C for 8 min, 40 cycles of 95 °C for 20 s, and 60 °C for 1 min. Fluorescence was read at the end of each round of the annealing step. Specimens that produced an increase in the fluorescence signal of the reporter dye over the threshold value (set at 10% of the maximum fluorescence of the positive amplification control), auto baseline, and produced a characteristic sigmoidal curve were considered to be positive for *M. bovis*. The DNA extracts from all the specimens were also tested for *M. bovis* by conventional PCR, and its sensitivity and specificity was compared to our Taqman assay. Conventional PCR was carried out in a T3 Thermocycler (Biometra- Göttingen, Germany), and the conditions used were as described by Gonzalez *et al.* [19]. Similarly, our Taqman assay was compared to the Taqman assay of Clothier *et al.* using their recipe and thermocycling conditions [12]. Table 1 provides sequence information of primers and probe used for conventional PCR as per Gonzalez *et.al.* [19] and Taqman PCR as per Clothier *et.al.* [12].

### 2.4. Culture

Culture was carried out directly from the clinical specimens in accordance with the protocol described previously by Goll and colleagues [2]. The specimens were processed as follows: (i) lung tissues: an incision was made on the tissue surface with a sterile scalpel blade. A sterile cotton tipped applicator was penetrated into the incised tissue to obtain the inoculum, which was then cultured on Hayflick’s mycoplasma agar plates; (ii) milk samples, joint fluids, and semen samples: a sterile cotton tipped applicator was dipped into the clinical specimen, and the inoculum was cultured on Hayflick’s mycoplasma agar plates; (iii) nasal swabs: they were directly plated onto Hayflick’s mycoplasma agar plates. The inoculated plates were incubated at 37 °C with 5% CO_2_ for one week and were examined under a dissecting microscope for mycoplasma growth every other day during the incubation. The characteristic “fried egg” like “centered” colony appearance was interpreted as positive culture for *M. bovis* [2].

### 2.5. Sensitivity and Specificity Analyses

The specificity of the *M. bovis* Taqman assay was assessed using genomic DNA from 22 different bacterial and *Mycoplasma* species that could potentially be encountered with mastitis, arthritis and pulmonary infections in ruminants (Table 2). A subset of clinical specimens (*n* = 71) were also subjected to culture-based detection of *M. bovis*. The limit of detection and analytical sensitivity of the real-time assay was determined using 10-fold serial dilutions of a culture of *M. bovis* strain ATCC 25523 in triplicate. The number of copies of *M. bovis* template DNA was calculated using the online resource “dsDNA copy number calculator” from URI Genomics and sequencing center (http://www.uri.edu/research/gsc/resources/cndna.html).

**Table 2:** *Mycoplasma* sp. and other bacterial species tested for specificity of the real-time PCR assay.

Number	Species	Strain	Real-Time PCR
1	*Mycoplasma bovis*	ATCC 25523	Positive
2	*Mycoplasma bovis*	ATCC 27369	Positive
3	*Mycoplasma bovis*	Field isolate	Positive
4	*Mycoplasma alkalescens*	ATCC 29103	Negative
5	*Mycoplasma canadense*	ATCC 29418	Negative
6	*Mycoplasma arginini*	ATCC 23838	Negative
7	*Mycoplasma bovigenitalium*	ATCC 27748	Negative
8	*Staphylococcus aureus*	Field isolate	Negative
9	*Streptococcus agalactiae*	Field isolate	Negative
10	*Escherichia coli*	Field isolate	Negative
11	*Corynebacterium pseudotuberculosis*	Field isolate	Negative
12	*Streptococcus uberis*	Field isolate	Negative
13	*Coagulase Negative Staphylococcus*	Field isolate	Negative
14	*Pseudomonas aeruginosa*	Field isolate	Negative
15	*Klebsiella pneumonia*	Field isolate	Negative
16	*Proteus mirabilis*	Field isolate	Negative
17	*Serratia marcescens*	Field isolate	Negative
18	*Entereococcus faecalis*	Field isolate	Negative
19	*Arcanobacterium pyogenes*	Field isolate	Negative
20	*Candida spp*	Field isolate	Negative
21	*Histophilus somnus*	Field isolate	Negative
22	*Salmonella spp*	Field isolate	Negative
23	*Bacillus cereus*	Field isolate	Negative
24	*Pasteurella multocida*	Field isolate	Negative
25	*Mannheimia haemolytica*	Field isolate	Negative

## 3. Results and Discussion

### 3.1. Limit of Detection

**Figure 1:**
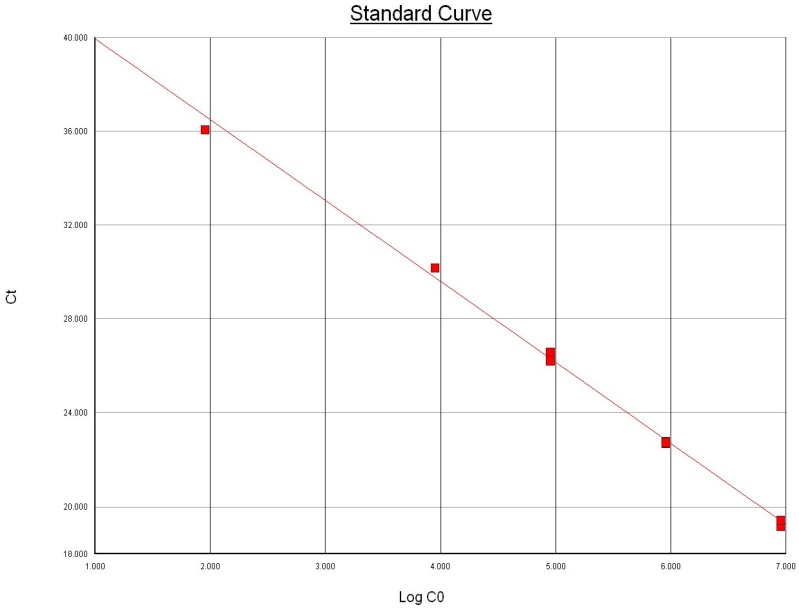
Relationship between the amount of template DNA copies (Log CO) in the Taqman assay and the mean threshold cycle (Ct). Slope: −3.448840; intercept: 43.393936; R^2^ = 0.996695.

Using 10-fold serial dilutions of genomic DNA extracted from pure culture of the international reference strain of *M. bovis* ATCC 25523 isolate, we determined the limit of detection for the *uvrC* gene-based Taqman assay similar to the method employed for *Bacillus anthracis* PCR by Moser *et al.* [20]. The extracted DNA was quantified and checked for purity using NanoDrop^®^ ND-1000 Spectrophotometer (Nanodrop Technologies, Inc. Wilmington, DE, USA). The DNA was tested in triplicate to determine the lowest detectable concentration, and the results indicated that the *Taqman assay* was able to detect 900 femtograms of total extracted DNA from all replicates (Figure 1). The genome length of *M. bovis* ATCC 25,523 was recently reported to be 1,003,404 bp [9]. Based on the observed Ct values and the standard curve, the assay demonstrated high sensitivity and was estimated to have a detection limit of ~83 copies of *uvrC* gene (Figure 1) and approximately 4 × 10^2^ cfu/mL. The limit of detection of our Taqman assay is comparable to the limit of detection reported by Clothier *et al.* [12].

### 3.2. Assay Specificity

The specificity of our Taqman assay was evaluated with genomic DNA from 22 bacterial and *Mycoplasma* species which could likely be present in infections from where *M. bovis* could be detected (Table 2). Both *M. bovis* ATCC isolates and a known clinical isolate of *M. bovis* were detected by the Taqman assay. No cross-reactivity was observed with other DNA targets, suggesting 100% specificity of the Taqman assay. The *uvrC* gene in *M. bovis* has been shown to be stable and resistant to normal mutation process reinforcing its efficacy as a target for PCR assays [16]. Thomas *et al.* [16] have demonstrated the *uvrC* gene to be a conserved target suitable for PCR based diagnostics by PCR amplification and sequencing from 92 and 20 *M. bovis* strains respectively. The specificity of our Taqman assay is similar to the specificity reported for another Taqman assay with *uvrC* target for *M. bovis* detection [12].

### 3.3. Assay Performance with Clinical Specimens

Analysis of the specimens from our study revealed *M. bovis* by Taqman PCR in 82 out of the 214 specimens (Table 3). Out of these 82 positives, the break-up was as follows: 93.3% of lung samples were positive (42 of 45), 19.2% of milk samples were positive (15 out of 78), 28.2% of nasal swabs were positive (22 of 78), 40% of joint fluid samples were positive (2 of 5), and 12.5% of semen specimens were positive (1 of 8). Although transmission of *M. bovis* through infected semen has been cited as a route of infection [1], to our knowledge, this is the first report of a PCR confirmed positive for *M. bovis* from semen.

The performance of our Taqman assay matched 100% (Table 3) with two previously published PCR methods: conventional PCR targeting the 16S rRNA [19] and Taqman real-time PCR targeting *uvrC* gene [12]. This study demonstrates our Taqman assay to have at least equivalent sensitivity and specificity as the other two PCR assays and that either of the two *uvrC* targeting Taqman assays could be utilized for the rapid and accurate detection of *M. bovis*.

**Table 3:** Comparison between our Taqman PCR assay, conventional PCR and Clothier *et al.* Taqman assay.

Specimen	Our Taqman PCR Assay		Conventional PCR		Clothier *et al*. Taqman PCR Assay	
	Positive	Negative	Positive	Negative	Positive	Negative
Lung (*n* = 45)	42	3	42	3	42	3
Milk (*n* = 78)	15	63	15	63	15	63
Nasal swab (*n* = 78)	22	56	22	56	22	56
Joint fluid (*n* = 5)	2	3	2	3	2	3
Semen (*n* = 8)	1	7	1	7	1	7
Total (*n* = 214)	82	132	82	132	82	132

*n* = number of specimen of each kind of specimen matrix tested.

### 3.4. Performance Comparison of Culture and Taqman PCR Assay

Gonzalez *et al.* has demonstrated that pre-enrichment does not significantly increase the isolation of *Mycoplasma* sp*.* but increases the cost of diagnosis based on their comparative study of over 4000 milk samples which were tested for *Mycoplasma* sp. by both: direct inoculation of milk on Hayflick agar plates and pre-enrichment in Hayflick broth before culturing on Hayflick agar plates [21]. Therefore, we directly cultured the clinical specimens without pre-enrichment in the Hayflick mycoplasma broth.

Out of the 214 clinical specimens, a subset (*n* = 71) was evaluated for the presence of *M. bovis* by culture examination which is cited in the literature as the gold standard test for *M. bovis* detection (Table 4). Interestingly, only 35.21% (*n* = 25) of these 71 specimens were positive for *M. bovi*s by culture method, whereas 70.42% (*n* = 50) were positive by our Taqman assay. Notably 27 out of the remaining 46 culture-negative specimens were positive by Taqman assay, while two specimens were positive by culture but negative by Taqman assay. Bell *et al.* recently reported similar findings and found PCR to detect 45.28% more of the positive *M. bovis* cases which were negative by the *M. bovis* culture test [22]. It is noteworthy that in addition to *M. bovis,* other bacterial and viral respiratory pathogens were also detected in some of the clinical specimens. However, in this study we did not investigate the concomitant *M. bovis* infections in presence or absence of these respiratory pathogens. The detection of *M. bovis* in clinical specimens may not always correlate with presence of an infectious underlying disease as *M. bovis* has been detected from both, clinically healthy animals and sick animals [23].

**Table 4:** Comparison between culture and our Taqman PCR assay.

Specimen	Culture		Our Taqman PCR Assay	
	Positive	Negative	Positive	Negative
Lung (*n* = 40)	17	23	39	1
Milk(*n* = 18)	4	14	4	14
Swabs(*n* = 10)	4	6	6	4
Joint Fluid (*n* = 3)	0	3	1	2
Total (*n* = 71)	25	46	50	21

Discrepancy between the outcome of culture results and Taqman assay results can be attributed to various factors. Due to the fastidious nature and fragility of *Mycoplasma* sp., suspected clinical specimens need to be promptly received within 24 h of sample collection in chilled condition to prevent loss of viability [7]. Whereas, PCR can detect *M. bovis* DNA from non-viable organisms and provides enhanced sensitivity compared to culture based examination [24]. Interestingly, Castillo-Alcala and colleagues have observed a large number of samples to be culture-positive for *M. bovis*, but were negative for *M. bovis* by hybridization probes based real-time PCR assay on a LightCycler platform [23]. Sensitivity of the PCR assays for *M. bovis* detection thus varies amongst different platforms of PCR. Isolation of *M. bovis* is significantly impaired from antibiotic treated animals and especially from lungs of chronically affected animals. Also co-infection, bacterial overgrowth, and contamination with other bacteria hinders with *M. bovis* isolation. Culture allows growth of all kinds of *Mycoplasma* species, and it is not specific for *M. bovis* detection as is the case with Taqman assay. Species level identification of *M. bovis* is not solely possible based on culture morphology, but requires further testing for confirmation by ELISA, PCR or immuno-histochemistry [7]. Inclusion of an internal extraction/ amplification control is recommended as it will assist in identifying any false negative results. As the internal control co-amplifies with the *uvrC* target gene, it serves in detecting any possible PCR inhibitors derived from the specimen DNA [25].

## 4. Conclusions

The *uvrC* gene-based Taqman real-time PCR assay described in this study is much faster than conventional PCR and culture based identification of *M. bovis*. The Taqman assay results can be obtained within 2.5 h with greater sensitivity and specificity in identifying *M. bovis* DNA from clinical specimens. We have validated our *uvrC* gene based Taqman real-time PCR assay to serve as a rapid and accurate test for the detection of *M. bovis* from a variety of clinical specimens.
